# UPAR silencing reveals its role in neuroblastoma

**DOI:** 10.18632/oncotarget.25821

**Published:** 2018-07-27

**Authors:** Natalia Kalinina

**Affiliations:** Department of Biochemistry and Molecular Medicine, Faculty of Medicine, Lomonosov Moscow State University, Moscow, Russia

**Keywords:** uPAR, neuroblastoma, trkC, CRISPR/Cas9

Receptor of urokinase-type plasminogen activator (uPAR) is the one of key players in cancer initiation and progression. uPAR interaction with its specific ligand urokinase (uPA) promotes cancer cell migration, invasion, and metastasis; whereas its lateral interaction with integrins and other transmembrane receptors, including EGFR and IGFR1, leads to initiation of intracellular signaling responsible for the activation of dormant cancer cells, epithelial-mesenchymal transition as well as enhanced survival and resistance to apoptosis of tumor cells. Pro-survival signaling activated by uPAR might be responsible for the escape of cancer cells from the cytotoxic effects of anticancer drugs [1]. uPA-uPAR interaction could also be responsible for tumor stroma activation [2] and tumor angiogenesis [3].

These multifaceted roles of uPAR in cancers make it an attractive target for anti-cancer therapy. Various approaches to inhibit uPAR expression or activity, including small molecules, antibodies and siRNAs demonstated significant down-regulation of tumor growth due to reduced tumor vascularization, suppressed cancer cell survival and proliferation. Although suppression of uPAR by siRNA is effective, it is not stable and therefore may not be sufficient due to the rapid dilution of siRNAs in fast proliferating cancer cells [4].

Recent study by Rysenkova et al. scheduled for publishing in Oncotarget provides novel insights into the role of uPAR in neuroblastoma. Earlier, elevated expression of uPAR was found in human neuroblastoma and correlated with high invasiveness of this tumor [5]. In the current study authors employed the CRISPR/Cas9n genome-editing technology to target the uPAR-encoding gene (*Plaur*) in Neuro 2A neuroblastoma cells. Down-regulation of uPAR expression caused a reduction in proliferation of neuroblastoma cells accompained by their enhanced apoptosis. These data indicate that CRISPR/Cas9n technology could be feasible for uPAR silencing in neuroblastoma [6].

Importantly, authors demonstrate that uPAR silencing correlated with a decrease in full-length TrkC mRNA expression, which could not be accounted for off-target effect of applied method. TrkC belongs to the family of tropomyosin-related kinase receptors, comprised of TrkA, TrkB and TrkC itself. These are transmembrane receptors with tyrosine kinase activity, which specifically bind neurotrophic growth factors. TrkC interaction with its ligand neurotrophin-3 (NT-3) provides pro-survival and proliferating stimuli for neural cells. However in the absence of NT-3 vacant TrkC induces apoptosis, behaving as a classical «dependence receptor». Such apoptosis activation in the absence or dramatic drop down of the ligand is important mechanism of limiting cell numbers during development and could be beneficial in tumorogenesis. Indeed, high expression of TrkC is associated with favorable outcome for neuroblasoma patients [7]. Pro-survival action of TrkC is associated with Akt and p38MAPK phosphorylation. Rysenkova et al. demonstrated that uPAR silencing is accompained by the prominent down-regulation in Akt and p38MAPK phosphorylation. These data indicate that TrkC-mediated pro-survival and proliferating stimuli in Neuro 2A cells depend on the presence of uPAR.

These data also indicate that urokinase system is likely to be involved in the control of neural cell sensitivity to NT-3. Previously uPAR was shown to be involved in the control of neuronal cell migration and directed axonal growth [8]. Data obtained by Rysenkova et al., further expand the role of uPAR in neural cell physiology and provide novel insights in its participation in the development of neuronal malignancies.
